# Association of endothelin genetic variants and hospitalized infection complications in end-stage renal disease (ESRD) patients

**DOI:** 10.1186/s12882-019-1349-3

**Published:** 2019-06-05

**Authors:** Chih-Chin Kao, Shih-Ying Cheng, Yu-Jia Wang, Shu-Chen Chien, Yu-Wen Hsu, Mei-Yi Wu, Hsing-Fang Lu, Sean Nam, Tao Sun, Mai-Szu Wu, Wei-Chiao Chang

**Affiliations:** 1 Graduate Institute of Clinical Medicine, College of Medicine, Taipei Medical University, Taipei, Taiwan; 2 Division of Nephrology, Department of Internal Medicine, Taipei Medical University Hospital, Taipei, Taiwan; 3 Division of Nephrology, Department of Internal Medicine, School of Medicine, College of Medicine, Taipei Medical University, Taipei, Taiwan; 4 Department of Pharmacy, Taipei Medical University Hospital, Taipei, Taiwan; 5 Department of Clinical Pharmacy, School of Pharmacy, College of Pharmacy, Taipei Medical University, Taipei, Taiwan; 6 Ph.D. Program for Neural Regenerative Medicine, College of Medical Science and Technology, Taipei Medical University, Taipei, Taiwan; 7 Clinical Research Center, Taipei Medical University Hospital, Taipei, Taiwan; 8 Ph.D. Program for Translational Medicine, College of Medical Science and Technology, Academia Sinica, Taipei Medical University, Taipei, Taiwan; 9 Division of Nephrology, Department of Internal Medicine, Shuang Ho Hospital, Taipei Medical University, New Taipei City, Taiwan; 10 Department of Surgery, University of Chicago, Chicago, IL USA; 11 Department of Pharmacy, Wan Fang Hospital, Taipei Medical University, Taipei, Taiwan; 12 Master Program for Clinical Pharmacogenomics and Pharmacoproteomics, School of Pharmacy, Taipei Medical University, Taipei, 110 Taiwan; 13 Department of Medical Research, Shuang Ho Hospital, Taipei Medical University, New Taipei City, Taiwan

**Keywords:** Endothelin, End stage renal disease, Infection, Renal failure

## Abstract

**Background:**

Infection is the second most common cause of mortality for patients with end-stage renal disease (ESRD), accompanying with immune dysfunction. Endothelin (*EDN*) is known to be related to inflammation; however, it is unknown whether genetic variants of the *EDN* gene family are associated with increased risk of hospitalized infection events.

**Methods:**

Nineteen tagging single-nucleotide polymorphisms (tSNPs) of the *EDN* gene family were selected for genotyping a cohort of 190 ESRD patients. Patient demographics were recorded, the subtypes of infection events were identified, and association analysis between the *EDN* genetic variants and hospitalized infection events was performed.

**Results:**

In this study, 106 patients were hospitalized for infection events. The leading events were pneumonia, bacteremia, and cellulitis. The minor allele of rs260741, rs197173, and rs926632 SNPs of *EDN3* were found to be associated with reduced risk of hospitalized bacteremia events.

**Conclusions:**

The minor allele of rs260741, rs197173, and rs926632 in *EDN3* were associated with reduced risk of hospitalized bacteremia events in ESRD patients.

**Electronic supplementary material:**

The online version of this article (10.1186/s12882-019-1349-3) contains supplementary material, which is available to authorized users.

## Background

Infection is known as a common cause of morbidity and mortality in patients with end-stage renal disease (ESRD), accounting for 20% of total deaths in these patients [1]. More than half of patients hospitalized for infection events developed an unfavorable outcome, including prolonged hospitalization, intensive care unit (ICU) stay, or even death [2, 3]. Impaired innate and adaptive immunity are associated with increased risk of infection in patients with renal failure [4]. A handful of cytokines was dysregulated in ESRD patients; for example, the production of interleukin (IL)-1, tumor necrosis factor (TNF)-α, and IL-6 was increased, whereas the bioavailability of IL-2 was reduced [5].

Genetic variants of immune-related genes have been shown to reflect risk of infection. Ferwerda et al. reported that the genetic variants of Toll-like receptor 4, which underlies the differential production of cytokines may affect the immune system, which in turn influenced the susceptibility of infection events and the risk of gram-negative bacterial infection [6]. Moreover, another study demonstrated that an *IL-9* variant may influence the susceptibility of respiratory syncytial virus infection [7].

Endothelin-1 (ET-1) is the major isoform of the endothelin (*EDN*) family and is a potent vasoconstrictor. It is associated with proinflammatory cytokine production, fibrosis, and angiogenesis [8]. The release of lipopolysaccharide from bacteria impairs the integrity of the endothelial cell, resulting in endothelial cell injury and cytokine release [9]. The plasma ET-1 level is increased during sepsis and is correlated with the severity of sepsis [10]. ET-1 is known to increase reactive oxygen species (ROS) production, and associates with activation of nuclear factor-kappaB and inflammatory cytokines such as TNF-α, IL-1, and IL-6 [11]. In addition, Lin et al. reported that ET-1 is able to increase cyclooxygenase-2 expression and prostaglandin E2 release [12]. These findings suggest that ET-1 is involved in the inflammatory reactions and the severity of sepsis. Regarding ET-3, Sato et al. demonstrated that low level of ET-3 reduces inflammatory responses [13]. However, it is still unclear whether *EDN* genetic variants are associated with hospitalized infection events in ESRD patients. In this study, genetic association study was applied to investigate the correlations between *EDN* genetic variants and the hospitalized infection events in ESRD patients.

## Methods

### Study population

Patients who received dialysis for more than 3 months at Taipei Medical University Hospital between September 2013 and June 2014 were enrolled. Demographic and laboratory data of these patients was shown in Table 1. The erythropoietin resistance index (ERI) was determined to characterize patients’ response to erythropoietin. ERI was calculated by dividing the weekly body-weight-adjusted epoetin dose by the hemoglobin concentration. Kt/V was used to measure dialysis adequacy. Outpatient and discharge medical records were used to determine the cause of ESRD. We prospectively followed up with the patients who were hospitalized for infection events. This study was approved by the Institutional Review Board of Taipei Medical University (Approval No. 201309026). A written informed consent form was obtained from all patients.

**Table 1:** Baseline characteristics of study patients stratified by infection events

	Infection events (*n* = 106)	No infection events (*n* = 84)	*P* value
Sex: Male, n (%)	58 (54.7%)	45 (53.6%)	0.875
Age (years)	66 ± 13	62 ± 13	**0.020**
Dialysis vintage (years)	4.6 ± 4.0	4.9 ± 6.1	0.705
Current smoking (%)	16 (15.1%)	9 (10.7%)	0.375
Diabetes, n (%)	55 (51.9%)	35 (41.7%)	0.161
ERI (unit/week/kg/Hb)	8.2 ± 6.9	8.6 ± 4.1	0.670
Hemoglobin (g/dL)	10.7 ± 1.3	10.8 ± 1.0	0.764
Albumin (g/dL)	3.9 ± 0.4	4.0 ± 0.4	**0.033**
Ferritin (mg/dL)	510 ± 664	390 ± 431	0.153
Iron (mg/dL)	66 ± 24	68 ± 31	0.610
TIBC (mg/dL)	231 ± 40	247 ± 54	**0.024**
Serum iPTH (pg/mL)	280 ± 349	390 ± 377	0.050
Kt/V	1.52 ± 0.30	1.48 ± 0.28	0.459
Cause of ESRD, n (%)			0.253
Hypertension	21 (19.8%)	16 (19.0%)	
Diabetes	54 (50.9%)	33 (39.3%)	
GN	18 (17.0%)	20 (23.8%)	
CHF	5 (4.7%)	3 (3.6%)	
Others	8 (7.5%)	12 (14.3%)	
Infection events			
Bacteremia	24 (22.6%)		
Pneumonia	38 (35.8%)		
UTI	10 (9.4%)		
Cellulitis	22 (20.8%)		
Peritonitis	21 (19.8%)		
IAI	10 (9.4%)		

The *P* values of < 0.05 are shown in bold

*Abbreviations*: *CHF* congestive heart failure, *ERI* erythropoietin resistance index, *GN* glomerulonephritis, *IAI* intra-abdominal infection, *iPTH* parathyroid hormone, *TIBC* total iron binding capacity, *UTI* urinary tract infection

### Infection events

We defined hospitalized “infection events” as bacteremia, pneumonia, cellulitis, urinary tract infection (UTI), peritonitis, and intra-abdominal infection (IAI). Bacteremia was confirmed by a positive blood culture result, and contamination was excluded by an infection specialist. Pneumonia was defined as the presence of clinical respiratory symptoms and the findings of increased infiltration on chest radiography. Cellulitis was determined as inflammation of the skin and subcutaneous tissues. UTI was defined as the presence of clinical symptoms and the detection of a pathogen in the urine. Peritonitis was diagnosed through clinical symptom examination and peritoneal fluid analysis. IAI was confirmed by the finding of intramural inflammation of the gastrointestinal (GI) tract without anatomic disruption. These diagnoses were ascertained by physicians and recorded in the discharge medical records.

### Genotyping

**Fig. 1:**
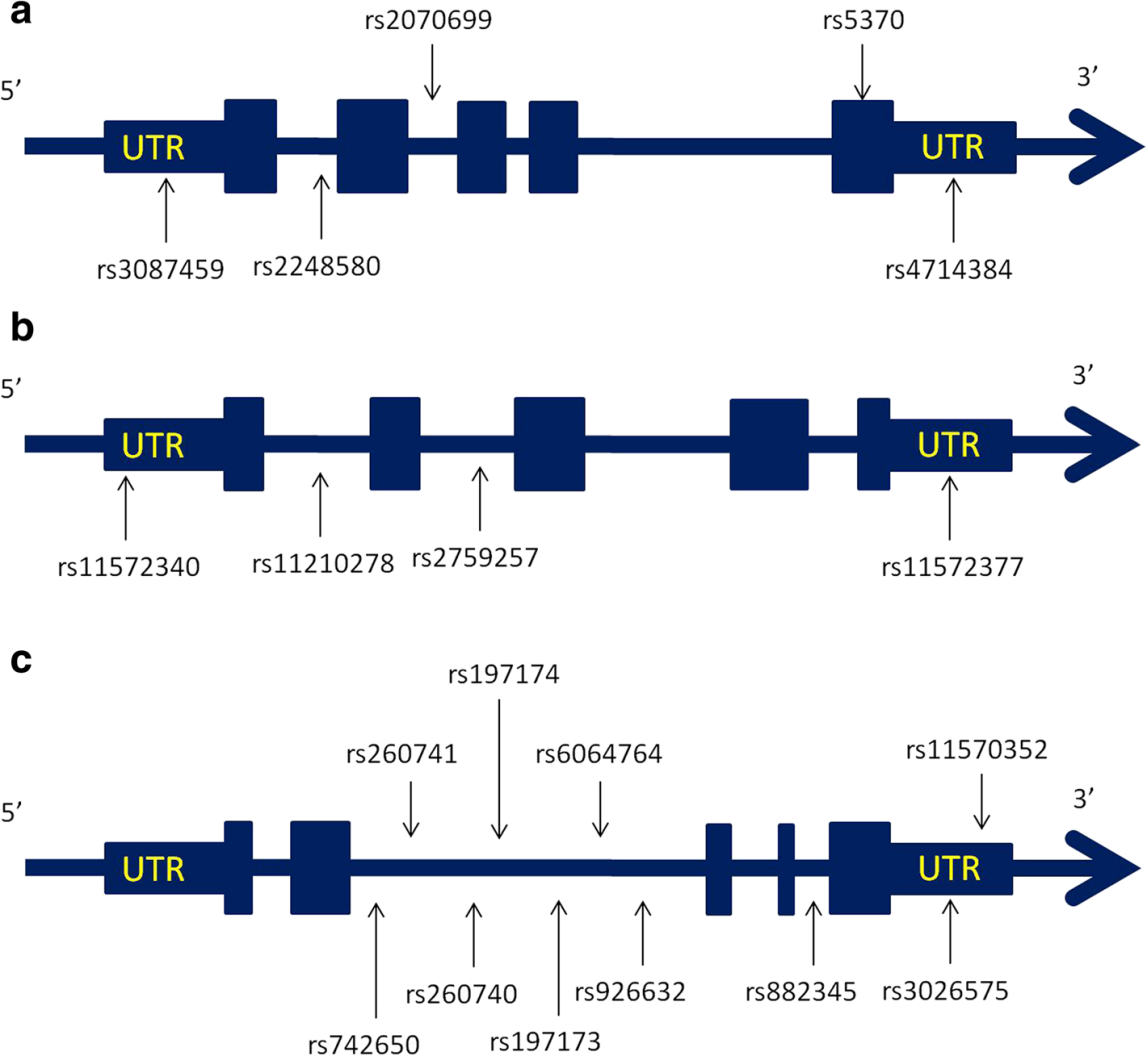
**a** Graphic view of the genotyped human *EDN1* gene. **b** Graphic view of the genotyped human *EDN2* gene. **c** Graphic view of the genotyped human *EDN3* gene

Patients’ blood samples were collected, and their genomic DNA was extracted. We selected 5 tagging single-nucleotide polymorphisms (tSNPs) of *EDN1* (i.e., rs5370, rs2070699, rs2248580, rs4714384, and rs3087459; Fig. 1a), 4 tSNPs of *EDN2* (i.e., rs2759257, rs11210278, rs11572340, and rs11572377*;* Fig. 1b), and 10 tSNPs of *EDN3* (i.e., rs742650, rs260740, rs260741, rs6064764, rs197173, rs197174, rs882345, rs926632, rs3026575, and rs11570352; Fig. 1c). The SNPs were selected by using r^2^ > 0.8 for linkage disequilibrium (LD) and MAF > 10% in a Beijing Han Chinese population as the setting of the Haploview software 4.1 (Broad Institute, Cambridge, MA, USA). The SNPs that are located in exon or untranslated regions (UTR) are defined as high priority targets to be included in selection. The LD map of target genes and SNPs selection were shown based on r^2^ and D’ in Additional file 1: Figures S1-S3. We performed genotyping using a TaqMan Allelic Discrimination Assay (Applied Biosystems, Foster City, CA). A polymerase chain reaction was performed on an ABI StepOnePlus Thermal Cycler (Applied Biosystems, Foster City, CA). The fluorescence from different probes was detected and analyzed using System SDS software version 2.2.2 (Applied Biosystems, Foster City, CA).

### Statistical analysis

Statistical analyses were conducted using R 3.2.0 (http://www.r-project.org/; http://cran.r-project.org/). The chi-squared test and Student’s *t* test were used to compare demographic characteristics between study (infection events) and control (no infection events) group. *P* < 0.05 was considered statistically significant. We used a multivariable logistic regression model to analyze the association of the *EDN* SNPs with hospitalized infection events. Four genetic models (genotype, dominant, recessive, allelic model) were evaluated in this study. These models were adjusted to reduce the confounding effects, including age, sex, diabetes, hemoglobin, albumin, and the cause of ESRD. LD was analyzed, and haplotype blocks were drawn using the default setting of the Haploview software 4.1 (Broad Institute, Cambridge, MA, USA).

## Results

### Clinical characteristics of patients

A total of 190 ESRD patients were enrolled at Taipei Medical University Hospital. Patient’s clinical characteristics were summarized in Table 1. More than half of patients (56%) developed hospitalized infection events during the observation period. The laboratory results haven’t displayed a significant difference between infection and non-infection groups, except for the albumin level and total iron binding capacity (TIBC). The cause of ESRD was found to be similar for both groups; diabetes mellitus was the main cause. The main hospitalized infection events were pneumonia (35.8%), followed by bacteremia (22.6%), cellulitis (20.8%), peritonitis (19.8%), UTI (9.4%), and IAI (9.4%). Patient’s demographics were stratified by bacteremia events, and six bacteria species, which are *Staphylococcus aureus*, Acinetobacter baumannii, *Escherichia coli*, *Enterococcus faecium*, Klebsiella pneumonia, and Staphylococcus hemolyticus, were under investigation (Additional file 1: Table S1). The inflammatory markers include procalcitonin (PCT) and c-reactive protein (CRP). The mean level of PCT was 3.6 ± 3.2 ng/mL, and CRP was 8.5 ± 9.2 mg/dL.

### Associations between *EDN* genetic variants and hospitalized infection events

The association analysis was performed for detecting the relationship of *EDN* genetic variants and hospitalized infection events. We haven’t observed a significant association between the frequencies of both *EDN1* and *EDN2* genotypes and clinical outcomes (Additional file 1: Table S2 and S3), but the rs882345, rs926632 and rs3026575 of *EDN3* showed a modest association with hospitalized infection events (Table 2).

**Table 2:** Analysis of association between *EDN3* single-nucleotide polymorphisms (SNPs) and hospitalized infection events

	Genotype	Infection (*n* = 106)	(%)	Without infection (*n* = 84)	(%)	Genotype model	Dominant model	Recessive model	Allelic model
						*P* value	*P* value	*P* value	*P* value
rs742650	TT	1	1.2	1	1.7	0.4215	0.8156	0.2224	0.9896
	CT	18	20.9	9	15.5				
	CC	67	77.9	48	82.8				
rs260740	GG	0	0.0	1	1.7	0.3925	0.8894	0.1864	0.9252
	GT	29	30.9	14	23.3				
	TT	65	69.1	45	75.0				
rs260741	AA	6	7.1	6	11.1	0.5823	0.4068	0.3609	0.3127
	AG	30	35.3	20	37.0				
	GG	49	57.6	28	51.9				
rs6064764	CC	2	2.1	1	1.3	0.1387	0.0568	0.9998	0.0938
	CT	21	22.1	7	9.3				
	TT	72	75.8	67	89.3				
rs197173	TT	6	6.7	9	13.8	0.5902	0.9957	0.3368	0.6674
	GT	31	34.8	20	30.8				
	GG	52	58.4	36	55.4				
rs197174	GG	2	2.1	3	4.9	0.2504	0.2100	0.1494	0.1253
	GA	17	18.1	11	18.0				
	AA	75	79.8	47	77.0				
rs882345	GG	4	4.3	0	0.0	**0.0168**	0.2688	**0.0350**	0.7671
	GA	9	9.7	12	18.5				
	AA	80	86.0	53	81.5				
rs926632	CC	3	3.2	2	2.9	**0.0096**	**0.0095**	0.6393	0.0534
	CT	9	9.5	18	25.7				
	TT	83	87.4	50	71.4				
rs3026575	AA	0	0.0	1	1.5	0.0754	0.2642	**0.0269**	0.1212
	AG	6	6.4	5	7.7				
	GG	88	93.6	59	90.8				
rs11570352	TT	3	2.9	5	6.0	0.3515	0.7034	0.2324	0.4364
	TC	4	3.8	1	1.2				
	CC	97	93.3	78	92.9				

The *P* value was adjusted for age, sex, diabetes, hemoglobin, albumin, and the cause of ESRD. The *P* values of < 0.05 are shown in bold

### Associations between *EDN* genetic variants and hospitalized bacteremia events

The rs260741, rs6064764, rs197173, and rs926632 of *EDN3* genotypes showed a significant association with hospitalized bacteremia events (Table 3). Patients carrying the minor allele of *EDN3* rs260741 (AA/AG vs. GG), rs197173 (TT/TG vs. GG), and rs926632 (CC/CT vs. TT) had a lower risk of hospitalized bacteremia events (genotype model, dominant model, and allelic model *P* value < 0.05). The minor allele of rs6064764 (CC/CT vs. TT) was associated with increased risk of bacteremia according to the dominant model and allelic model *P* values (Table 3). No association of *EDN1* or *EDN2* SNPs with hospitalized bacteremia events were detected (Additional file 1: Table S4 and S5).

**Table 3:** Analysis of association between *EDN3* single-nucleotide polymorphisms (SNPs) and hospitalized bacteremia events

	Genotype	Bacteremia (*n* = 24)	(%)	Without bacteremia (*n* = 84)	(%)	Genotype model	Dominant model	Recessive model	Allelic model
						*P* value	*P* value	*P* value	*P* value
rs742650	TT	0	0	1	1.7	0.4983	0.3770	0.5773	0.4870
	CT	6	30.0	9	15.5				
	CC	14	70.0	48	82.8				
rs260740	GG	0	0.0	1	1.7	0.5886	0.9802	0.3185	0.8360
	GT	7	33.3	14	23.3				
	TT	14	66.7	45	75.0				
rs260741	AA	0	0.0	6	11.1	**0.0203**	**0.0165**	**0.0350**	**0.0077**
	AG	4	21.1	20	37.0				
	GG	15	78.9	28	51.9				
rs6064764	CC	1	4.5	1	1.3	0.0905	**0.0286**	0.5282	**0.0447**
	CT	6	27.3	7	9.3				
	TT	15	68.2	67	89.3				
rs197173	TT	0	0.0	9	13.8	**0.0447**	**0.0393**	**0.0465**	**0.0188**
	GT	4	19.0	20	30.8				
	GG	17	81.0	36	55.4				
rs197174	GG	0	0.0	3	4.9	0.3176	0.3178	0.1659	0.2094
	GA	5	25.0	11	18.0				
	AA	15	75.0	47	77.0				
rs882345	GG	0	0.0	0	0.0	0.1100	NA	NA	NA
	GA	1	4.8	12	18.5				
	AA	20	95.2	53	81.5				
rs926632	CC	0	0.0	2	2.9	**0.0395**	**0.0123**	0.3256	**0.0112**
	CT	1	4.8	18	25.7				
	TT	20	95.2	50	71.4				
rs3026575	AA	0	0.0	1	1.5	0.8634	0.5878	0.9999	0.5879
	AG	1	4.8	5	7.7				
	GG	20	95.2	59	90.8				
rs11570352	TT	1	4.2	5	6.0	0.6086	0.4834	0.6859	0.5661
	TC	0	0.0	1	1.2				
	CC	23	95.8	78	92.9				

The *P* value was adjusted for age, sex, diabetes, hemoglobin, albumin, and the cause of ESRD. The *P* values of < 0.05 are shown in bold

### Haplotypes analysis of *EDN3* with infection and bacteremia events

**Fig. 2:**
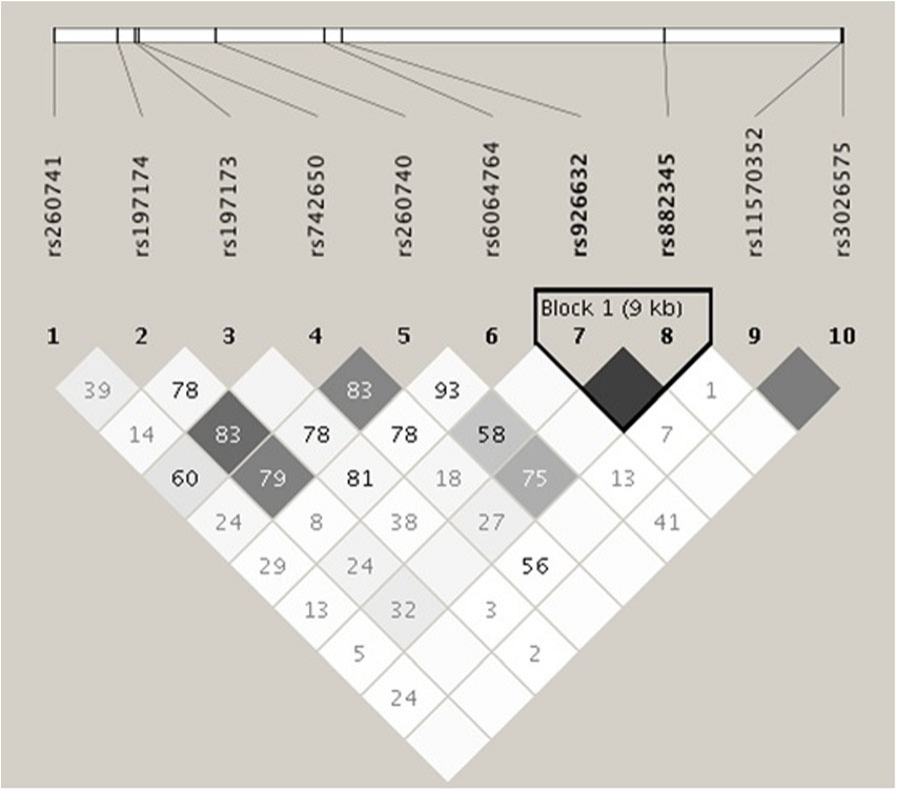
*EDN3* gene linkage disequilibrium (LD) and haplotype block structure in ESRD patients. The number on the cell is the LOD score of r^2^

Next, we further validated the effects of haplotypes of *EDN3* gene by pairwise linkage disequilibrium (LD) (Fig. 2). The haplotype frequency of *EDN3* rs926632/rs882345 variants among patients with hospitalized infection and bacteremia events were shown in Tables 4 and 5. The *EDN3* haplotype had no significant association with the risk of infection or bacteremia events. To test the possible functional roles of the polymorphisms in *EDN3*, we queried expression quantitative trait loci (eQTL) of *EDN3* via Genotype-Tissue Expression (GTEx) database in different types of tissues [14]. Of note, the low expression level of EDN3 was found in immune cells (Additional file 1: Figure S4). In addition, we analyzed the SNPs to understand the functional roles through using HaploReg V4.1. Interestingly, results indicated that the non-coding SNP rs260741 and rs6064764 of *EDN3* were potentially related to the regulation of T cells activation by epigenetic modifications [15].

**Table 4:** Haplotype frequencies of *EDN3* gene rs926632/rs882345 among patients with hospitalized infection events or not

rs926632/rs882345	Case	Control	OR (95% CI)	*P* value
T/A	91%	84%	1	Ref
C/A	NA	6%	–	**–**
C/G	9%	9%	0.93(0.46–1.90)	0.8131

Haplotype frequency less than 1% was excluded

*Abbreviation*: *OR* odds ratio

**Table 5:** Haplotype frequencies of *EDN3* gene rs926632/rs882345 among patients with hospitalized bacteremia events or not

rs926632/rs882345	Case	Control	OR (95% CI)	*P* value
T/A	97%	87%	1	Ref
C/A	NA	3%	–	–
C/G	3%	10%	0.29 (0.04–2.02)	0.1791

Haplotype frequency less than 1% was excluded

*Abbreviation*: *OR* odds ratio

## Discussion

Dialysis patients are exposed to high risk of infection, and related mortality [16, 17]. Ishigami et al. reported that a low estimated glomerular filtration rate and high albuminuria were associated with increased risk of hospitalization for infection, including bloodstream infections, pneumonia, UTI, and cellulitis [18]. Among these infections, bacteremia was the most critical and responsible for three-quarters of infection-related mortality [19]. The risk factors for infection in dialysis patients include age, immunosuppressive therapy, poor hygiene, and low performance status [17, 20–22]. Age, albumin level, and category of infection have been shown to associate with poor outcomes [23]. Old age and lower albumin level were the risk factors for infection events. Indeed, both innate and adaptive immunity are known to change with age, which may result in a persistent low-grade inflammation and tissue damage [24]. Furthermore, T-cell repertoire (TCR) complexity was revealed to predict the EPO responsiveness. Thus, TCR repertoire diversity may indicate the immune responses to infection [25]. Malnutrition, inflammation, and atherosclerosis syndrome, characterized by patients with renal failure, are predictive of poor outcomes [26]. Importantly, malnutrition has also been reported to associate with chronic inflammation, followed by increased risk of infection [27].

Correa et al. reported that *EDN* signaling pathway is involved in the pathogenesis of mycobacteria tuberculosis infection, and that *EDN* receptor A or B signaling is critical for the host responses to the infection [28]. Wilson et al. indicated the functional roles of *EDN* genes in intestinal hypoperfusion during bacteremia [29]. Additionally, the precursor peptide ET-1 of *EDN*1 gene is correlated with the severity of pneumonia, ICU admission, and mortality [30]. Also, ET-1 has been shown to stimulate ROS production [9] and correlate with the risk of inflammatory diseases such as atherosclerosis. Our previous study showed genetic variants of *EDN1* gene are associated with an increased risk of hospitalization for a cardiovascular event [31]. Pittet et al. demonstrated that the serum level of ET-1 was strongly correlated with reduced cardiac output in sepsis patients [32]. In this study, we found the correlation between *EDN* genetic variants and hospitalized infection events..

A previous study on *EDN3* and EDN receptor type B (*EDNRB*) knockout mouse model showed that *EDN3* and *EDNRB* were associated with severe enterocolitis [33]. The ET-3 peptide of *EDN3* gene was reported to evoke an attenuated inflammation by inducing the EDN B2 receptor and nitric oxide production [13]. Another study also proposed that ET-3 reduces platelet-activating factor (PAF)-induced inflammation by directly binding to PAF [34]. Interestingly, the Human Protein Atlas showed that ET-3 is highly expressed in the GI tract and endocrine tissues [35]. During the development of enteric nervous system, ET-3 and its receptor, EDNRB, orchestrate the signaling cascades for enteric ganglion formation [36–38]. The enteric nervous system, composing of neurons and glial cells, have been shown to interact with the outer (microbiota) and inner environments (immune cells) [39]; the enteric microbiota is known to interact with the immune system [40]. These studies indicated that ET-3 may affect the inflammation process through the modification of enteric nervous system and interaction with the microbiota. The results provided indirect evidence for supporting our findings in this study.

This study has limitations. First, our small sample size may affect the statistical power in the analysis. Second, the gene expression and protein level of *EDN* were not measured from patients’ samples. Further validation studies are needed in order to evaluate the associations of genetic variants with gene/protein expression. Third, a replication study should be conducted in the second population.

## Conclusions

The minor allele of rs260741, rs197173, and rs926632 in *EDN3* was associated with a reduced risk of hospitalized bacteremia events in patients with end-stage renal disease (ESRD).

## Additional files

Additional file 1: **Figure S1.** Linkage disequilibrium (LD) map of *EDN1* gene SNPs shown (a) based on R-squared (r^2^) (b) based on D-prime (D’). **Figure S2.** Linkage disequilibrium (LD) map of *EDN2* gene SNPs shown (a) based on R-squared (r^2^) (b) based on D-prime (D’). **Figure S3.** Linkage disequilibrium (LD) map of *EDN3* gene SNPs shown (a) based on R-squared (r^2^) (b) based on D-prime (D’). **Figure S4.** *EDN3* gene expression across different tissues**. Table S1.** Baseline characteristics of study patients stratified by bacteremia events**. Table S2.** Analysis of association between *EDN1* single-nucleotide polymorphisms (SNPs) and hospitalized infection events. **Table S3.** Analysis of association between *EDN2* single-nucleotide polymorphisms (SNPs) and hospitalized infection events. **Table S4.** Analysis of association between *EDN1* single-nucleotide polymorphisms (SNPs) and hospitalized bacteremia events. **Table S5.** Analysis of association between *EDN2* single-nucleotide polymorphisms (SNPs) and hospitalized bacteremia events. (DOCX 196 kb)

## Abbreviations

EDN: Endothelin
ERI: Erythropoietin resistance index
ESRD: End-stage renal disease
ET-1: Endothelin-1
GTEx: Genotype-Tissue Expression
Kt/V: Adequacy of dialysis
