# Supplementary material for: Association of endothelin genetic variants and hospitalized infection complications in end-stage renal disease (ESRD) patients
Source: BMC Nephrol. 2019 Jun 5;20:203. doi: 10.1186/s12882-019-1349-3 (PMC6549338; doi:10.1186/s12882-019-1349-3)
Supplement: Supplementary file 1 — Figure S1. Linkage disequilibrium (LD) map of EDN1 gene SNPs shown (a) based on R-squared (r2) (b) based on D-prime (D’). Figure S2. Linkage disequilibrium (LD) map of EDN2 gene SNPs shown (a) based on R-squared (r2) (b) based on D-prime (D’). Figure S3. Linkage disequilibrium (LD) map of EDN3 gene SNPs shown (a) based on R-squared (r2) (b) based on D-prime (D’). Figure S4. EDN3 gene expression across different tissues. Table S1. Baseline characteristics of study patients stratified by bacteremia events. Table S2. Analysis of association between EDN1 single-nucleotide polymorphisms (SNPs) and hospitalized infection events. Table S3. Analysis of association between EDN2 single-nucleotide polymorphisms (SNPs) and hospitalized infection events. Table S4. Analysis of association between EDN1 single-nucleotide polymorphisms (SNPs) and hospitalized bacteremia events. Table S5. Analysis of association between EDN2 single-nucleotide polymorphisms (SNPs) and hospitalized bacteremia events. (DOCX 196 kb) [file 12882_2019_1349_MOESM1_ESM.docx]

**Figure S1. Linkage disequilibrium (LD) map of *EDN1* gene SNPs shown (a) based on R-squared (r^2^) (b) based on D-prime (D')**

(a)


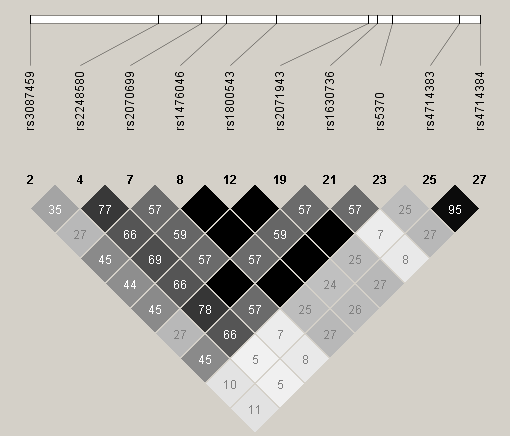


(b)


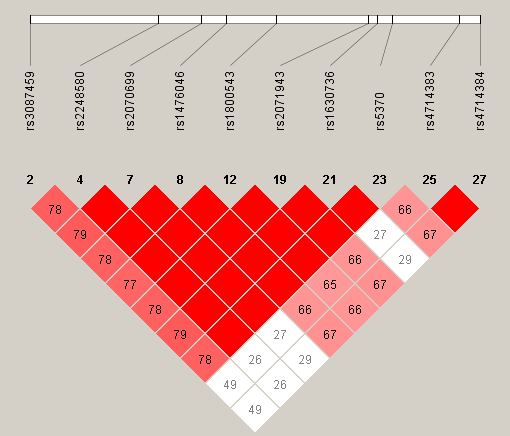


**Figure S2. Linkage disequilibrium (LD) map of *EDN2* gene SNPs shown (a) based on R-squared (r^2^) (b) based on D-prime (D')**

(a)


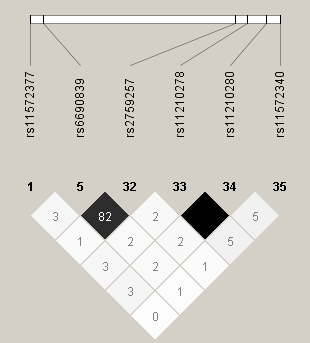


(b)


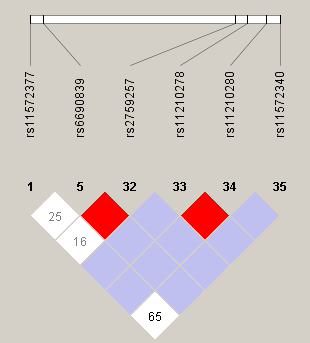


**Figure S3. Linkage disequilibrium (LD) map of *EDN3* gene SNPs shown (a) based on R-squared (r^2^) (b) based on D-prime (D')**

(a)


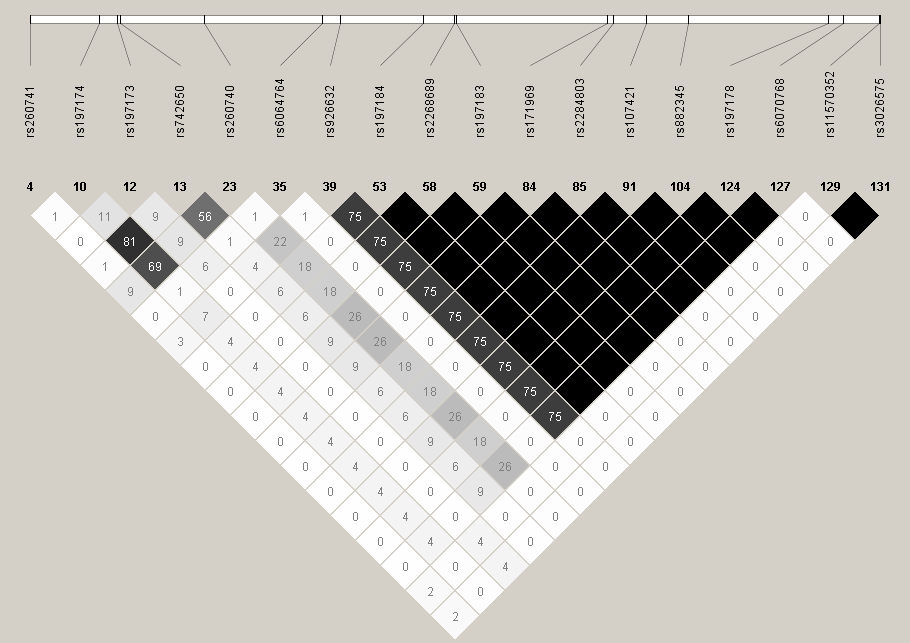


(b)


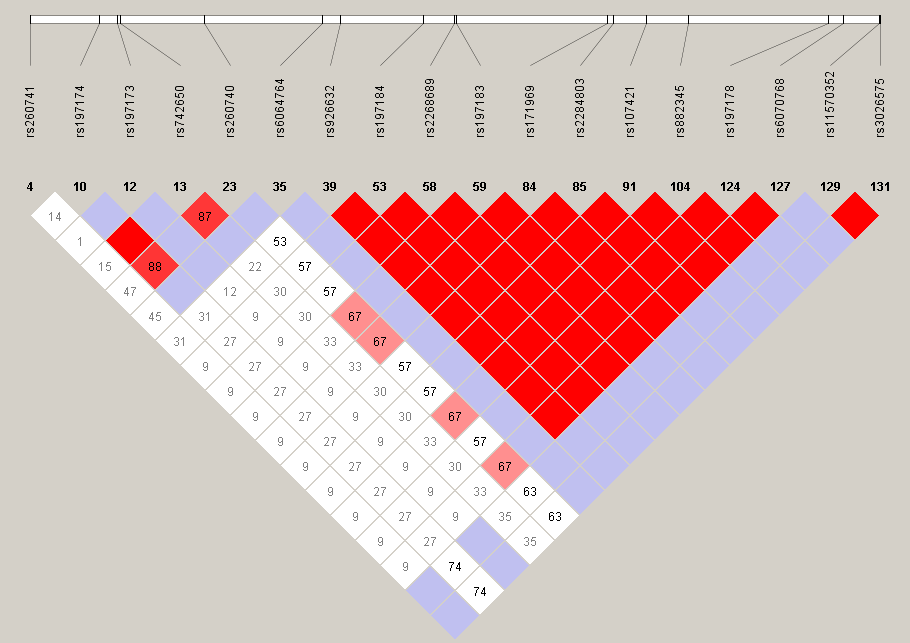


**Figure S4. *EDN3* gene expression across different tissues**


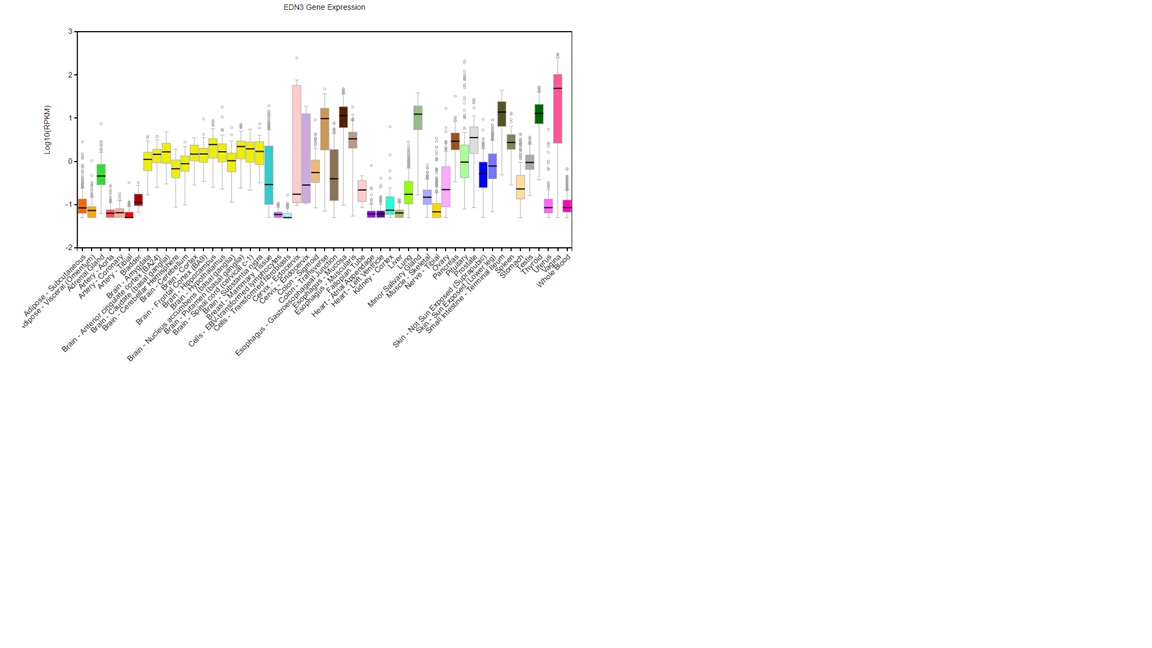


Vertical axis represents log reads per kilobase transcript per million (RPKM). These data were obtained from the GTEx Portal (https://gtexportal.org/home/) on June 23, 2017.

| **Table S1. Baseline characteristics of study patients stratified by bacteremia events** | | | |
| --- | --- | --- | --- |
|  | Bacteremia events  (n = 24) | Without bacteremia  (n = 84) | *P* value |
| Sex: Male, n (%) | 9 (37.5%) | 45 (53.6%) | 0.165 |
| Age (years) | 66 ± 13 | 62 ± 13 | 0.140 |
| Dialysis vintage (years) | 3.9 ± 3.3 | 4.9 ± 6.0 | 0.463 |
| Current smoking (%) | 3 (12.5%) | 9 (10.7%) | 0.806 |
| Diabetes, n (%) | 13 (54.2%) | 35 (41.7%) | 0.277 |
| ERI (unit/week/kg/Hb) | 8.8 ± 4.5 | 8.6 ± 4.1 | 0.856 |
| Hemoglobin (g/dL) | 10.5 ± 1.1 | 10.8 ± 1.0 | 0.366 |
| Albumin (g/dL) | 3.9 ± 0.6 | 4.0 ± 0.4 | 0.089 |
| Ferritin (mg/dL) | 583 ± 620 | 390 ± 431 | 0.092 |
| Iron (mg/dL) | 66 ± 24 | 67 ± 31 | 0.188 |
| TIBC (mg/dL) | 231 ± 40 | 247 ± 53 | 0.085 |
| Serum iPTH (pg/mL) | 171 ± 188 | 390 ± 377 | **0.016** |
| Kt/V | 1.48 ± 0.25 | 1.48 ± 0.28 | 0.976 |
| Cause of ESRD, n (%) |  |  | 0.866 |
| Hypertension | 5 (20.8%) | 16 (19.0%) |  |
| Diabetes | 13 (54.2%) | 33 (39.3%) |  |
| GN | 4 (16.7%) | 20 (23.8%) |  |
| CHF | 0 (0%) | 3 (3.6%) |  |
| Others | 2 (8.3%) | 12 (14.3%) |  |
| Bacteria species, n (%) |  |  |  |
| Staphylococcus aureus | 10 (41.7%) |  |  |
| Acinetobacter baumannii | 3 (12.5%) |  |  |
| Escherichia coli | 3 (12.5%) |  |  |
| Enterococcus faecium | 3 (12.5%) |  |  |
| Klebsiella pneumonia | 3 (12.5%) |  |  |
| Staphylococcus hemolyticus | 2 (8.3%) |  |  |
| Abbreviations: CHF: congestive heart failure; ERI: erythropoietin resistance index; GN: glomerulonephritis; iPTH: parathyroid hormone; TIBC: total iron binding capacity. The *P* value <0.05 are shown in bold. | | | |

| **Table S2. Analysis of association between *EDN1* single-nucleotide polymorphisms (SNPs) and hospitalized infection events** | | | | | | | | | |
| --- | --- | --- | --- | --- | --- | --- | --- | --- | --- |
|  | Genotype | Infection  (n = 106) | (%) | Without infection  (n = 84) | (%) | Genotype model | Dominant model | Recessive model | Allelic model |
|  |  |  |  |  |  | *P* value | *P* value | *P* value | *P* value |
| rs5370 | TT | 5 | 5.7 | 4 | 6.3 | 0.9895 | 0.8916 | 0.9245 | 0.8845 |
|  | TG | 30 | 34.5 | 22 | 34.9 |  |  |  |  |
|  | GG | 52 | 59.8 | 37 | 58.7 |  |  |  |  |
| rs2070699 | GG | 16 | 19.0 | 11 | 17.2 | 0.7776 | 0.5818 | 0.8086 | 0.8143 |
|  | GT | 42 | 50.0 | 30 | 46.9 |  |  |  |  |
|  | TT | 26 | 31.0 | 23 | 35.9 |  |  |  |  |
| rs2248580 | CC | 10 | 11.1 | 10 | 14.9 | 0.4765 | 0.5116 | 0.4668 | 0.9244 |
|  | CA | 44 | 48.9 | 27 | 40.3 |  |  |  |  |
|  | AA | 36 | 40.0 | 30 | 44.8 |  |  |  |  |
| rs4714384 | TT | 11 | 12.8 | 10 | 15.9 | 0.3444 | 0.1908 | 0.2772 | 0.1444 |
|  | TC | 34 | 39.5 | 25 | 39.7 |  |  |  |  |
|  | CC | 41 | 47.7 | 28 | 44.4 |  |  |  |  |
| rs3087459 | CC | 3 | 3.4 | 3 | 4.5 | 0.7344 | 0.8031 | 0.5454 | 0.9829 |
|  | CA | 23 | 26.4 | 16 | 24.2 |  |  |  |  |
|  | AA | 61 | 70.1 | 47 | 71.2 |  |  |  |  |
| The *P* value was adjusted for age, sex, diabetes, hemoglobin, albumin, and the cause of ESRD. The *P* value <0.05 are shown in bold. | | | | | | | | | |

| **Table S3. Analysis of association between *EDN2* single-nucleotide polymorphisms (SNPs) and hospitalized infection events** | | | | | | | | | |
| --- | --- | --- | --- | --- | --- | --- | --- | --- | --- |
|  | Genotype | Infection  (n = 106) | (%) | Without infection  (n = 84) | (%) | Genotype model | Dominant model | Recessive model | Allelic model |
|  |  |  |  |  |  | *P* value | *P* value | *P* value | *P* value |
| rs2759257 | AA | 0 | 0.0 | 0 | 0.0 | 0.3785 | NA | NA | NA |
|  | AC | 15 | 15.8 | 8 | 10.1 |  |  |  |  |
|  | CC | 80 | 84.2 | 71 | 89.9 |  |  |  |  |
| rs11210278 | TT | 7 | 10.1 | 8 | 16.3 | 0.3504 | 0.3204 | 0.5725 | 0.6548 |
|  | TC | 27 | 39.1 | 12 | 24.5 |  |  |  |  |
|  | CC | 35 | 50.7 | 29 | 59.2 |  |  |  |  |
| rs11572340 | AA | 0 | 0.0 | 1 | 1.2 | 0.3043 | 0.4980 | 0.1267 | 0.3112 |
|  | AC | 4 | 3.8 | 3 | 3.6 |  |  |  |  |
|  | CC | 101 | 96.2 | 80 | 95.2 |  |  |  |  |
| rs11572377 | GG | 2 | 1.9 | 1 | 1.2 | 0.3601 | 0.2099 | 0.9651 | 0.3308 |
|  | GC | 8 | 7.7 | 4 | 4.8 |  |  |  |  |
|  | CC | 94 | 90.4 | 78 | 94.0 |  |  |  |  |
| The *P* value was adjusted for age, sex, diabetes, hemoglobin, albumin, and the cause of ESRD. The *P* value <0.05 are shown in bold. | | | | | | | | | |

| **Table S4. Analysis of association between *EDN1* single-nucleotide polymorphisms (SNPs) and hospitalized bacteremia events** | | | | | | | | | |
| --- | --- | --- | --- | --- | --- | --- | --- | --- | --- |
|  | Genotype | Bacteremia  (n = 24) | (%) | Without  Bacteremia  (n = 84) | (%) | Genotype model | Dominant model | Recessive model | Allelic model |
|  |  |  |  |  |  | *P* value | *P* value | *P* value | *P* value |
| rs5370 | TT | 2 | 10.0 | 4 | 6.3 | 0.1812 | 0.0657 | 0.4701 | 0.0823 |
|  | TG | 1 | 55.0 | 22 | 34.9 |  |  |  |  |
|  | GG | 7 | 35.0 | 37 | 58.7 |  |  |  |  |
| rs2070699 | GG | 4 | 20.0 | 11 | 17.2 | 0.2804 | 0.1226 | 0.8996 | 0.3062 |
|  | GT | 13 | 65.0 | 30 | 46.9 |  |  |  |  |
|  | TT | 3 | 15.0 | 23 | 35.9 |  |  |  |  |
| rs2248580 | CC | 4 | 19.0 | 10 | 14.9 | 0.3072 | 0.1334 | 0.8153 | 0.2712 |
|  | CA | 12 | 57.1 | 27 | 40.3 |  |  |  |  |
|  | AA | 5 | 23.8 | 30 | 44.8 |  |  |  |  |
| rs4714384 | TT | 2 | 10.5 | 10 | 15.9 | 0.1548 | 0.8040 | 0.0905 | 0.5157 |
|  | TC | 10 | 52.6 | 25 | 39.7 |  |  |  |  |
|  | CC | 7 | 36.8 | 28 | 44.4 |  |  |  |  |
| rs3087459 | CC | 0 | 0 | 3 | 4.5 | 0.0448 | 0.1202 | 0.1212 | 0.3987 |
|  | CA | 9 | 24.2 | 16 | 24.2 |  |  |  |  |
|  | AA | 10 | 71.2 | 47 | 71.2 |  |  |  |  |
| The *P* value was adjusted for age, sex, diabetes, hemoglobin, albumin, and the cause of ESRD. The *P* value <0.05 are shown in bold. | | | | | | | | | |

| **Table S5. Analysis of association between *EDN2* single-nucleotide polymorphisms (SNPs) and hospitalized bacteremia events** | | | | | | | | | |
| --- | --- | --- | --- | --- | --- | --- | --- | --- | --- |
|  | Genotype | Bacteremia  (n = 24) | (%) | Without  Bacteremia  (n = 84) | (%) | Genotype model | Dominant model | Recessive model | Allelic model |
|  |  |  |  |  |  | *P* value | *P* value | *P* value | *P* value |
| rs2759257 | AA | 0 | 0.0 | 0 | 0.0 | 0.9650 | NA | NA | NA |
|  | AC | 3 | 14.3 | 8 | 10.1 |  |  |  |  |
|  | CC | 18 | 85.7 | 71 | 89.9 |  |  |  |  |
| rs11210278 | TT | 3 | 23.1 | 8 | 16.3 | 0.7675 | 0.9478 | 0.5794 | 0.8124 |
|  | TC | 3 | 23.1 | 12 | 24.5 |  |  |  |  |
|  | CC | 7 | 53.8 | 29 | 59.2 |  |  |  |  |
| rs11572340 | AA | 0 | 0.0 | 1 | 1.2 | 0.6702 | 0.6152 | 0.3870 | 0.4968 |
|  | AC | 1 | 4.2 | 3 | 3.6 |  |  |  |  |
|  | CC | 23 | 95.8 | 80 | 95.2 |  |  |  |  |
| rs11572377 | GG | 0 | 0 | 1 | 1.2 | 0.4246 | 0.4384 | 0.4674 | 0.6716 |
|  | GC | 2 | 8.3 | 4 | 4.8 |  |  |  |  |
|  | CC | 22 | 91.7 | 78 | 94.0 |  |  |  |  |
| The *P* value was adjusted for age, sex, diabetes, hemoglobin, albumin, and the cause of ESRD. The *P* value <0.05 are shown in bold. | | | | | | | | | |
